# Supplementary material for: Structural and Technological Characterization of Tropical Smallholder Farms of Dual-Purpose Cattle in Mexico
Source: Animals (Basel). 2020 Jan 5;10(1):86. doi: 10.3390/ani10010086 (PMC7023156; doi:10.3390/ani10010086)
Supplement: Supplementary file 1 [file animals-10-00086-s001.pdf]

Supplementary Metarials

Tabla S1. Typologies for dual-purpose farms.

Study	Farming classification	Author
Sinaloa, México	Four types of livestock farms by size; small (67 %), medium (24 %), big (7 %) and big with business potential (2 %).	[29]
Chiapas, Mexico	Classification by size. Number of heads in the herd	[7, 31]
Sinaloa, México	Farms classification by innovation levels: -High (25.6%); -Medium (36.4%); -Low (38%).	[25]
South of the State of Mexico	Classification by level of efficiency: -Group 1: Small and efficient farms; -Group 2: Medium and little efficient farms; -Group 3: Big and extensive farms but little efficient; -Group 4: Subsistence farms.	[13]
Las Choapas, Veracruz, Mexico.	Classification by objectives: -Business farms (3%); -Transition farms (17%); -Traditional farms (80%).	[25, 27]
Morelos, Mexico	Classification by number of heads: -Business farms over than 150 heads (7%); -Livestock transition 60 heads (18%); -Familiar agriculture farms 30 to 50 heads (19%); -Familiar livestock 15 to 30 heads (21%); -Livestock subsistence 0 to 15 heads (35%).	[28]
Veracruz, México	Classification by animal units (UA) per farm: -Traditional 37,97 UA; -Transition 142,7 UA; -Business 177,3 UA.	[30]
Tabasco, Mexico	Classification by surface (farms):-Cow-calf (55.5%) 56 ha; -Traditional (38.8%) 147 ha; -Improved DP (3.8%) 282 ha; -Sire production (1.9%) 462 ha.	[6]
Veracruz, Mexico	Classification by level of production: -Specialized (20 to 27 l/d); -Semispecialized (18 to 20 l/d); -Familiar (6 to 12 l/d); -Dual purpose (3 to 9 l/d).	[24]
Center of Chiapas, Mexico	Classification by number of heads and technological level: -Semiextensive 91.8 heads; -Semiintensive 57.7 heads; -Extensive 101.1 heads.	[5, 31]
Las Choapas, Veracruz, México	Classification by size (land): -Traditional group (80 ranches); -Transition group (17 ranches); -Bussines group (3 ranches).	[27]
District 008, Veracruz, Mexico	Classification by technological level and objectives: -Subsistence-traditional; -Commercial traditional; -Semi-technified; -Technified.	[3, 30]
Mexico	Classification by Region (North, Center, East) and genetic breeds (High, intermediate, low, unknown).	[8, 26]

Table S2. Innovations and areas in the dual-purpose system.

A1. Management	Information system of management and direct use of resources by grazing
1. Animal identification	0. Individual animals identification was not done; 1. Individual identification was
2. Record system	0. Record systems were not utilized; 1. Record systems were utilized
3. Breeding management	0. There was not a specific management breeding planning; 1. There was
4. Grazing native pasture	0. Cattle did not graze in native pasture lands; 1. Cattle grazed in native pasture
5. Grazing planting	0. Cattle did not graze in planted pasture lands; 1. Cattle grazed in planted pasture

6. Grazing of crop residues	0. Grazing of crop residues was not done; 1 Grazing of crop residues was done
7. Milking season	0. Cows are not regularly milked; 1. Cows are regularly milked
8. Type of milking	0. Hand milking was utilized; 1. Mechanical milking was utilized mainly
**A2. Feeding**	**Strategies for animal feeding applied by smallholders**
9. Green fodder	0. Green fodders were not used; 1. Green fodders were cultivated
10. Silage	0. Feeding with silages was not utilized; 1. Feeding with silages was utilized
11. Hay making	0. Cattle were not fed with haymaking or stubble; 1. Cattle were
12. Processed feed	0. Cattle were not fed with processed feed; 1. Cattle were
13. Concentrate making	0. Cattle were not fed with concentrate-making feed; 1. Cattle were
14. Molasses/urea	0. Cattle were not supplemented with molasses/urea; 1. Cattle were
15. Grains and oilseeds	0. Grains and oilseeds were not added to cattle diet; 1. They were added
16. Multi nutritional blocks processed	0. Cattle were not supplemented with multi nutritional blocks processed; 1. Cattle were supplemented
17. Manufacture of multi nutritional blocks	0. Cattle were not supplemented with manufactured multi nutritional blocks; 1. Cattle were supplemented
18. Common salt	0. Cattle were not supplemented with NaCl; 1. Cattle were supplemented
19. Mineral salts	0. Cattle were not supplemented with mineral salts; 1. Cattle were supplemented
20. Mineral blocks	0. Cattle were not supplemented with mineral blocks; 1. Cattle were supplemented
21. Vitamin provided	0. Vitamins were not used; 1. Vitamins were provided, as A, D, E, B complex
22. Agro-industrial by-products	0. Agro-industrial by-products were not used; 1. Agro-industrial by-products awerere used.
**A3. Genetics**	**Technologies to improve productive parameters**
23. Using male breeds	0. Male breeds were not utilized; 1. Male breeds were incorporated
24. Using male crosses	0. Male crosses were not utilized; 1. Male crosses were incorporated
25. Using female breeds	0. Female breeds were not utilized; 1. Female breeds were incorporated
26. Using female crosses	0. Female crosses were not utilized; 1. Female crosses were incorporated
27. Use of genetically tested bulls	0. Genetically tested bulls were not utilized; 1. Genetically tested bulls were utilized
28. Calves selection criteria	0. Calves selection criteria were not used; 1. Calves selection criteria were used
29. Female selection criteria	0. Female selection criteria were not used; 1. Female selection criteria were used
30. Sire selection criteria	0. Sire selection criteria were not used: 1. Sire selection criteria were used
31. Crossbred system	0. Crossbreed planning was not utilized; 1. Crossbreed planning was utilized
**A4. Reproduction**	**Technologies oriented to improve reproductive efficiency parameters**
32. Evaluation in bulls	0. No evaluation of the reproductive capacity of bulls or no sire on the farm; 1. Evaluation of the reproductive capacity of bull is done
33. Semen evaluation	0. Sperm viability was not done; 1. Sperm fertility was evaluated
34. Female evaluation	0. Evaluation of female body condition was not done; 1. Evaluation was done
35. Oestrus detection,	0. Estrus detection was not done; 1. Estrus detection was done
36. Pregnancy Diagnosis	0. Pregnancy diagnosis was not done; 1. Pregnancy diagnosis was done
37. Mating	0. Seasonal mating; 1. Continuous mating was done
38. Breeding policy	0. Control of the mating was not done; 1. Planning mating control
**A5. Animal Health**	**Technologies geared to health, welfare, quality of the milk production**

39. Health planning	0. Animal health planning was not done; 1. Animal health planning was done.
40. Vaccination program	0. Planning of vaccines and bacterins was not done; 1. Application was done.
41. Parasite diagnosis	0. Diagnosis analysis was not utilized; 1. Diagnosis analysis was used.
42. Internal deworming control	0. Internal deworming was not used; 1. Internal deworming was used
43. External parasite control	0. External parasite control was not used; 1. External parasite control was used
44. Mastitis diagnosis	0. Mastitis diagnosis was not done; 1. Mastitis diagnosis was done
45. Sanitary milking program	0. A sanitary milking program was not done; 1. Were done
